# Efficacy of sorafenib, a multi-tyrosine kinase inhibitor, in an adenoid cystic carcinoma metastatic to the lung: case report and review of literature

**DOI:** 10.1186/1752-1947-5-483

**Published:** 2011-09-27

**Authors:** Daniel J Dammrich, Edgardo S Santos, Luis E Raez

**Affiliations:** 1 University of Miami/Sylvester Comprehensive Cancer Center, Miami, Florida, USA

## Abstract

**Introduction:**

Treatment for squamous cell carcinoma of the head and neck has significantly improved with the addition of cetuximab, a monoclonal antibody against the epidermal growth factor receptor, to conventional cytotoxic agents. The most significant aspect of this treatment approach is the proof that head and neck cancers are suitable for targeted therapies as has been shown in other malignancies. Unfortunately, there are other rare histologic types of head and neck cancer such as adenocarcinoma and adenoid cystic carcinoma. The latter has traditionally been considered to be chemotherapy resistant and surgical resection with or without adjuvant radiation therapy has been the rule as far as treatment is concerned. The course of adenoid cystic carcinoma ranges from indolent to aggressive; however, most patients succumb to the disease as a result of distant metastases. This clinical scenario poses a challenge to oncologists. Several conventional chemotherapy regimens and novel targeted agents have been tried in this rare histologic subtype without success.

**Case presentation:**

In this case report, we present a 59-year-old Caucasian female with refractory adenoid cystic carcinoma of the maxilla metastatic to the lung that responded to sorafenib, a novel multi-tyrosine kinase inhibitor, which targets angiogenesis, Raf kinase pathway, platelet-derived growth factor Ret, and c-Kit.

**Conclusion:**

This case illustrates the possibility that this chemoresistant tumor may need the inhibition or blocking of several oncogenic pathways. Certainly, it is imperative that more studies are done in this special population trying to identify tumorigenesis mechanisms that may be upregulated in this malignancy and could be potential targets for therapeutic development.

## Introduction

Adenoid cystic carcinoma (ACC) of the head and neck is an uncommon malignancy that accounts for approximately 10% of all neoplasms of the salivary glands [1]. While the parotid gland is the most common site of origin for these tumors, they can occur in any of the major or minor salivary glands. The major salivary glands include the parotid, submandibular and sublingual, and the minor salivary glands can be found throughout the oral and nasal cavities and in the paranasal sinuses. On histological analysis, ACC has three recognized growth patterns: cribriform, tubular and solid.

These tumors are usually slow growing and have a propensity for local recurrence, perineural invasion and, eventually, distant metastasis, especially in the lung and bones. A paranasal location, perineural invasion, solid histology, high stage, and positive margins after surgery all predict worse overall survival (OS). Although OS in these patients is reported as quite high at five years (50-90%), the 10- and 15-year survival rates are much lower (25-67%) [2]. In addition, once metastatic lesions are identified, the average survival is between 20 and 32 months depending on the location of the metastasis [1].

Radical excision followed by radiotherapy is generally considered to be the initial therapy of choice in patients with ACC [2]. Despite these efforts, local recurrence and distant metastasis occur frequently. Unfortunately, the role of chemotherapy and targeted agents is still poorly defined in these patients. Several chemotherapy agents and targeted therapies have been studied, however most of these have been looked at in small retrospective studies and the results have usually been disappointing.

The aim of this case report is to present a patient with metastatic ACC who achieved disease stabilization on sorafenib, after experiencing disease progression on two different lines of palliative chemotherapy. Sorafenib is a multi-tyrosine kinase inhibitor (mTKI), which is approved as first-line therapy for renal cell carcinoma, hepatoma and gastrointestinal stromal tumors. To the best of our knowledge there are no reports of the use of sorafenib in patients with metastatic ACC.

## Case presentation

**Figure 1:**
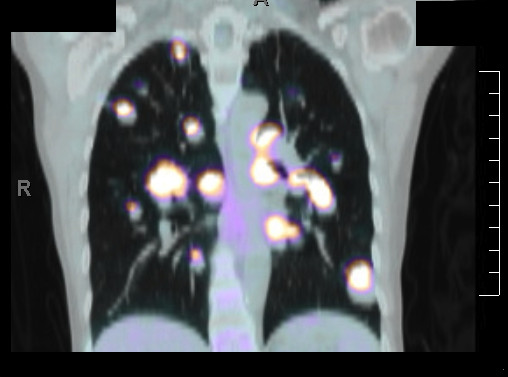
**PET/CT scan demonstrating metastatic disease before any systemic therapy was initiated**.

**Figure 2:**
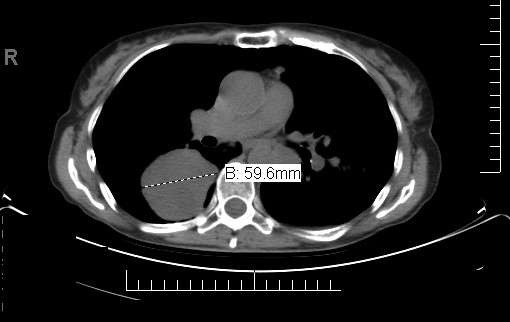
**PET/CT scan demonstrating metastatic disease before any systemic therapy was initiated**.

A 59-year-old Caucasian female presented five years prior to the presentation of this case report with symptoms of chronic sinusitis and, upon biopsy, was found to have ACC in her right inferior turbinate and right maxillary sinus tissue. The work-up for metastatic disease at that time, including computed tomography with positron emission tomography (CT/PET), was negative. She underwent surgical resection of this neoplasm shortly after diagnosis and two months later received adjuvant radiation therapy for one month. She did not experience any signs or symptoms of local recurrence or distant metastasis until a surveillance CT/PET scan was performed three years after her initial presentation (Figures 1 and 2), which revealed multiple bilateral lung masses. A biopsy of one of her right lung lesions confirmed the diagnosis of metastatic ACC. At this time, these lesions were deemed unresectable and she was referred to medical oncology for palliative chemotherapy.

**Figure 3:**
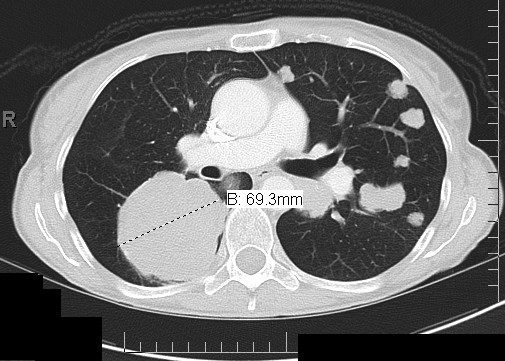
**CT scan demonstrating progression of disease after first line palliative chemotherapy with the CAP regimen**.

**Figure 4:**
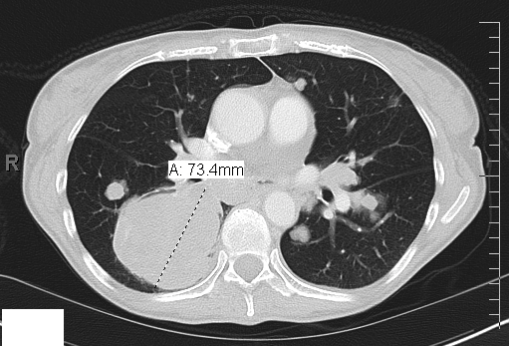
**CT scan demonstrating progression of disease after second line palliative chemotherapy with carboplatin and docetaxel**.

**Figure 5:**
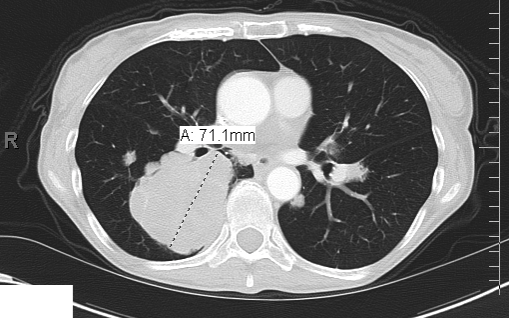
**CT scan demonstrating stable disease after five months of sorafenib**.

Our patient was initiated on palliative chemotherapy with CAP regimen (cisplatinum 50 mg/m^2^, Adriamycin (doxorubicin) 50 mg/m^2^, and cyclophosphamide 500 mg/m^2^) for six cycles. She experienced progression of the disease on therapy (Figure 3) and was switched to second-line therapy combining carboplatin (area under the concentration curve = 5) and docetaxel (75 mg/m^2^). She was preparing to start cycle three of carboplatin and docetaxel, however she was found to be pancytopenic, including grade three thrombocytopenia. The third cycle of chemotherapy was postponed and she was transfused with packed red blood cells and placed on growth factor support. During this delay restaging was performed, and once again she had experienced progression of the disease (Figure 4). After failing two lines of palliative chemotherapy, she was initiated on sorafenib at 400 mg orally twice a day. Sorafenib was chosen in this setting because it is an easily administered oral medication with a favorable side effect profile and has a wide variety of molecular targets. A surveillance CT/PET scan performed after two cycles of this therapy revealed stable disease, a clinical response that could not be attained with prior conventional cytotoxic agents (Figure 5). This response was based on the Response Evaluation Criteria in Solid Tumors (RECIST) [3]. According to RECIST, stable disease is defined as neither partial response, which is a 30% decrease in the sum of the two longest diameters of the tumor, nor progressive disease, which is a 20% increase in the sum of the two longest diameters of the tumor. Our patient did require a dose reduction of sorafenib of 12.5% due to the fact that she experienced hand-foot syndrome, which consists of reddening, swelling, numbness and desquamation on the palms and soles that can occur after chemotherapy in cancer patients. She also experienced occasional epistaxis. Otherwise, our patient has tolerated sorafenib well and continues on it due to disease stabilization for six months at the time of writing.

## Discussion

Sorafenib, an mTKI, is used in the treatment of advanced renal cell carcinoma and hepatocellular carcinoma. It has been shown to have activity against Raf kinase, vascular endothelial growth factor (VEGF) receptor 2, platelet derived growth factor receptor, Flt-3, Ret and c-Kit. Based on our review of the literature, we did not find any cases of ACC treated successfully with sorafenib.

The ideal treatment for metastatic ACC remains elusive; several single agents and combined chemotherapy regimens have been studied. Evidence suggests that single agent activity is strongest with platinum, 5-fluorouracil and anthracyclines, and that the most consistent response rates are achieved in combination with platinum, anthracyclines and alkylating agents [4]. Unfortunately, this response is often short-lived, as it was in this patient. Due to these results, there is no standard therapy for recurrent ACC [5].

The poor response of ACC to conventional cytotoxic agents has driven research into targeted molecular therapies, in the hope that they may provide more benefit for patients with metastatic ACC. *C-Kit *proto-oncogene, epidermal growth factor receptor (EGFR), human epidermal receptor 2 proto-oncogene, androgen receptors, p53 protein and VEGF are examples of recent targets that have been evaluated with varying degrees of expression in ACC. Kit is a transmembrane cell surface receptor encoded by the *c-Kit *proto-oncogene. Its role is to regulate cell growth, differentiation, and migration, and it is expressed in 65% to 100% of ACC [4]. Because of its overexpression, it was thought that imatinib, a TKI with activity against the c-Kit receptor, could provide benefit in patients with ACC. Unfortunately, a prospective study which included 16 ACC patients treated with imatinib demonstrated no response to this therapy [6]. Another study showed that imatinib might actually promote tumor progression in ACC [7].

Another potential target is the Her-2 receptor; its expression in ACC has been shown to be extremely variable with reports of anywhere from 4% to 100% of patients. The use of trastuzumab, a monoclonal antibody directed against the Her-2 receptor, has also been explored. Unfortunately, the results with this therapy have also been disappointing. A phase II study that enrolled 14 patients with advanced salivary gland tumors demonstrated partial response with trastuzumab in only one patient with mucoepidermoid carcinoma. The two patients in this study with ACC did not experience any benefits [8]. Other potential targets such as EGFR and VEGF have also been found to be variable in ACC.

## Conclusion

Targeted therapy against molecular targets has not been sufficiently evaluated in patients with ACC. To date, ACC has shown poor response to targeted therapies. In our case, our patient attained clinical benefit from sorafenib by obtaining a truly stable disease after twice progressing on active therapy. Sorafenib may have potential for activity in these tumors because it targets several different cellular pathways, and since it appears that these tumors are extremely variable in terms of the molecules they express, a broader approach to these targets seems to be reasonable.

Our patient appears to have classic tumor behavior for ACC; presence of metastatic disease and chemoresistance. The disease stabilization attained by our patient with single agent sorafenib is exciting. It also demonstrates that more research must been done to understand the molecular mechanisms that are involved in ACC and the potential targets for future therapy development.

## Consent

Written informed consent was obtained from the patient for publication of this case report and any accompanying images. A copy of the written consent is available for review by the Editor-in-Chief of this journal.

## Competing interests

The authors declare that they have no competing interests.

## Authors' contributions

LR and ES analyzed and interpreted the patient data, imaging, and response to treatment. DD was a major contributor in writing the manuscript. All authors read and approved the final manuscript.
